# Interferon-β and interleukin-6 exert opposing effects on Foxp3 acetylation to control regulatory T cell induction

**DOI:** 10.3389/fimmu.2025.1593931

**Published:** 2025-05-30

**Authors:** Mehek Ningoo, Francisco Fueyo-González, Cayetana Gisbert-Vilanova, Laura Espinar-Barranco, Nada Marjanovic, Miguel Fribourg

**Affiliations:** ^1^ Translational Transplant Research Center, Division of Nephrology, Department of Medicine, Icahn School of Medicine, New York, NY, United States; ^2^ Immunology Institute, Icahn School of Medicine, New York, NY, United States; ^3^ Center for Translational Medicine and Pharmacology, Department of Pharmacological Sciences, Icahn School of Medicine, New York, NY, United States; ^4^ Department of Neurology, Icahn School of Medicine, New York, NY, United States

**Keywords:** interferon-beta, interleukin-6, regulatory T cells, acetylation, tocilizumab

## Abstract

Cytokines are key soluble signaling molecules that regulate immune responses. With the advent of therapies that selectively target cytokines and cytokine receptors, understanding the molecular mechanisms underpinning how immune cells integrate multiple cytokine signals has become a critical challenge in immunology. However, the pleiotropic nature of cytokines makes it difficult to decipher their precise contributions in various contexts. Here, we used an integrated experimental and computational approach to investigate the combined effect and interplay between the pro-inflammatory cytokine interleukin-6 (IL-6) and interferon-beta (IFNβ), also a pro-inflammatory cytokine with potent antiviral properties, in modulating regulatory T cell (Treg) induction. Our studies reveal that, in contrast with its pro-inflammatory role in innate immune responses, IFNβ can counteract the well-described inhibitory effect of IL-6 on Treg induction. Mechanistically, we demonstrate that IFNβ and IL-6 signal independently to promote opposing effects on the acetylation of Foxp3, an essential transcription factor governing Treg differentiation, stability, and function. We further show that this mechanism is conserved in both murine and human T cells, highlighting the broad relevance of this finding in immune regulation. These results have important implications for the numerous contexts in which IFNβ and IL-6 co-exist, including viral infection, transplantation, and autoimmune disease.

## Introduction

Interferon beta (IFNβ) and interleukin-6 (IL-6) are pivotal pleiotropic cytokines that orchestrate immune responses, and whose dysregulation is implicated in various diseases (1–5). Both IFNβ and IL-6 share the ability to trigger and amplify an inflammatory response in a time, context, and cell type-specific matter (6–10). IFNβ is a type I interferon produced in response to viral infections exhibiting potent antiviral and pro-inflammatory properties. Additionally, IFNβ contributes to inflammation by responding to damage-associated molecular patterns (DAMPs) released from injured or dying cells (11). IL-6, a potent inflammatory cytokine, stimulates the liver to produce acute-phase proteins, induces fever, and enhances the inflammatory response by promoting neutrophil production and stimulating other pro-inflammatory cytokines (3). Given its central role in inflammation, IL-6 has become a prime therapeutic target, with IL-6 and IL-6 receptor inhibitors being FDA-approved. While IL-6 is acknowledged as a crucial mediator bridging innate and adaptive immunity, IFNβ has been historically associated with innate immune responses, and its role in modulating adaptive immune responses remains less explored.

Treg cells are important agents in maintaining peripheral and central immune tolerance (12–14), key immune processes in transplant and autoimmune disease, which are also exploited by tumors in cancer (15, 16). IL-6 reduces peripherally induced Treg in part by directly acting on naïve CD4^+^ T cells to favor their polarization to a pro-inflammatory helper 17 phenotype (Th17) (17–19). In contrast with its pro-inflammatory effects, our group previously reported that IFNβ can act directly on naïve CD4^+^ to enhance Treg cell induction by promoting Foxp3 acetylation through a molecular mechanism that is dependent on phosphorylated STAT1 (pSTAT1) signaling and the activity of the acetyltransferase P300 (20). This dichotomy and the common co-occurrence of IL-6 and IFNβ in numerous disease contexts, such as autoimmune disease and viral infection, has prompted the question in the field of how these two signals are integrated at the time of T cell polarization.

Here, we explored whether IFNβ could counteract the inhibitory effects of IL-6 on Treg cell induction. Our findings indicate that while IL-6 promotes deacetylation of Foxp3 to inhibit Treg formation, IFNβ can prevent this process by inducing a pro-acetylation transcriptional program. Our results support that IL-6 and IFNβ exert these opposing effects on Foxp3 acetylation through independent signaling mechanisms, highlighting the potential synergistic effects of IFNβ with therapeutic strategies targeting IL-6, currently being explored in the clinic.

## Materials and methods

A detailed list of reagents is provided in **Supplementary Table S1**.

### Mice

**Figure 1:**
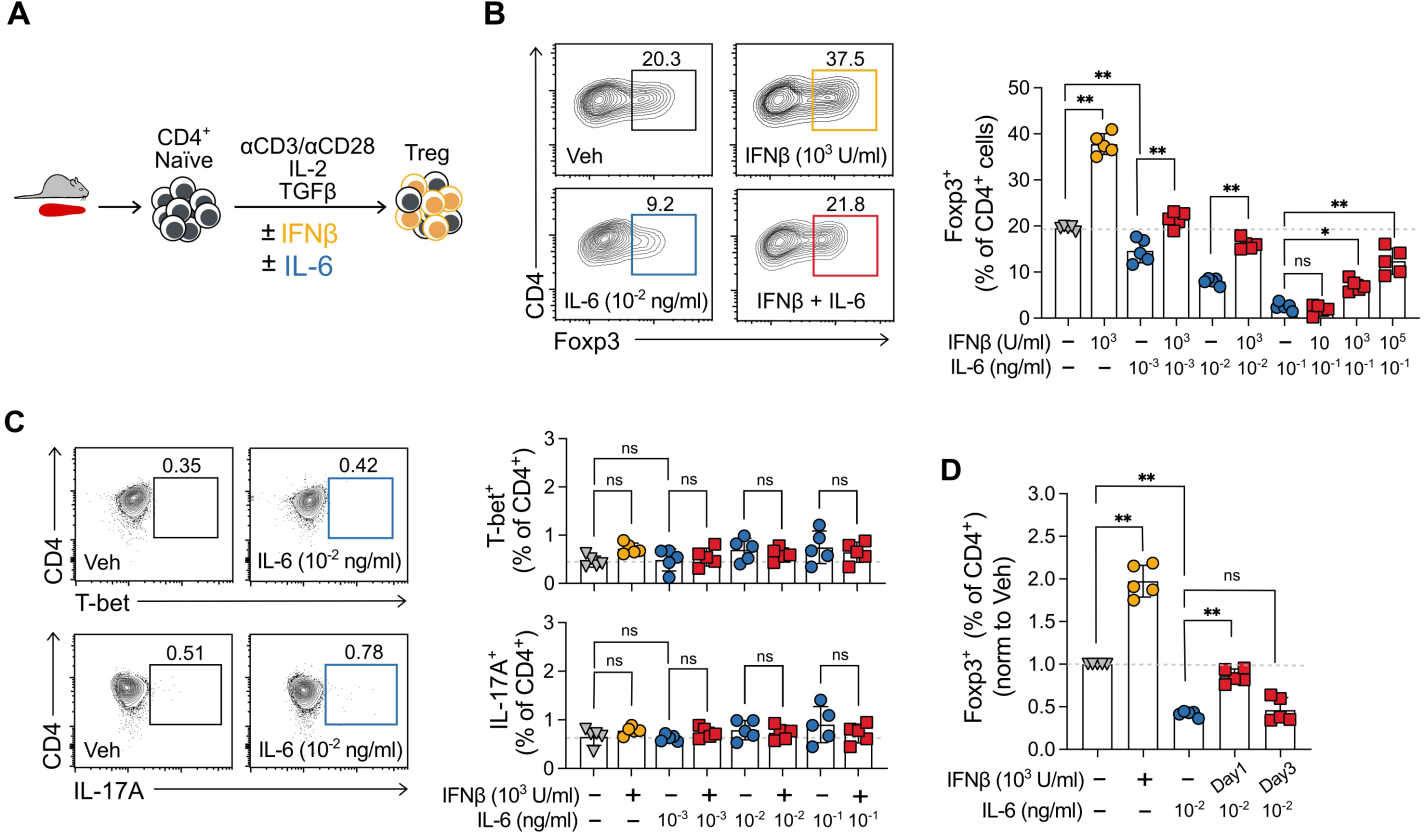
IFNβ prevents inhibition of Treg cell induction by IL-6. **(A)** Description of experimental Treg cell induction cultures from murine splenocytes in the presence of IL-6 and IFNβ. **(B)** Representative scatter plots and summary results of the percentage of Foxp3^+^ cells obtained at the end of 5-day Treg cell induction cultures when treated with IFNβ (10, 1000 or 100,000 U/ml), IL-6 (0.01, 0.1, or 1 ng/ml), IFNβ + IL-6, or vehicle **(C)** Representative scatter plots and summary results of Th1 and Th17 cells obtained at the end of the same Treg cell induction cultures in **(B)** when treated with IFNβ (1000 U/ml), IL-6 (0.01, 0.1, or 1 ng/ml), IFNβ + IL-6, or vehicle. **(D)** Summary results for identical Treg cell induction cultures as those in **(B)** in which IFNβ was added on day 0 or day 3 of the 5-day cultures. Summaries depict mean ± SD, ANOVA with *post hoc* Tukey HSD test, *p<0.05, **p<0.01, ns, not significant.

**Figure 2:**
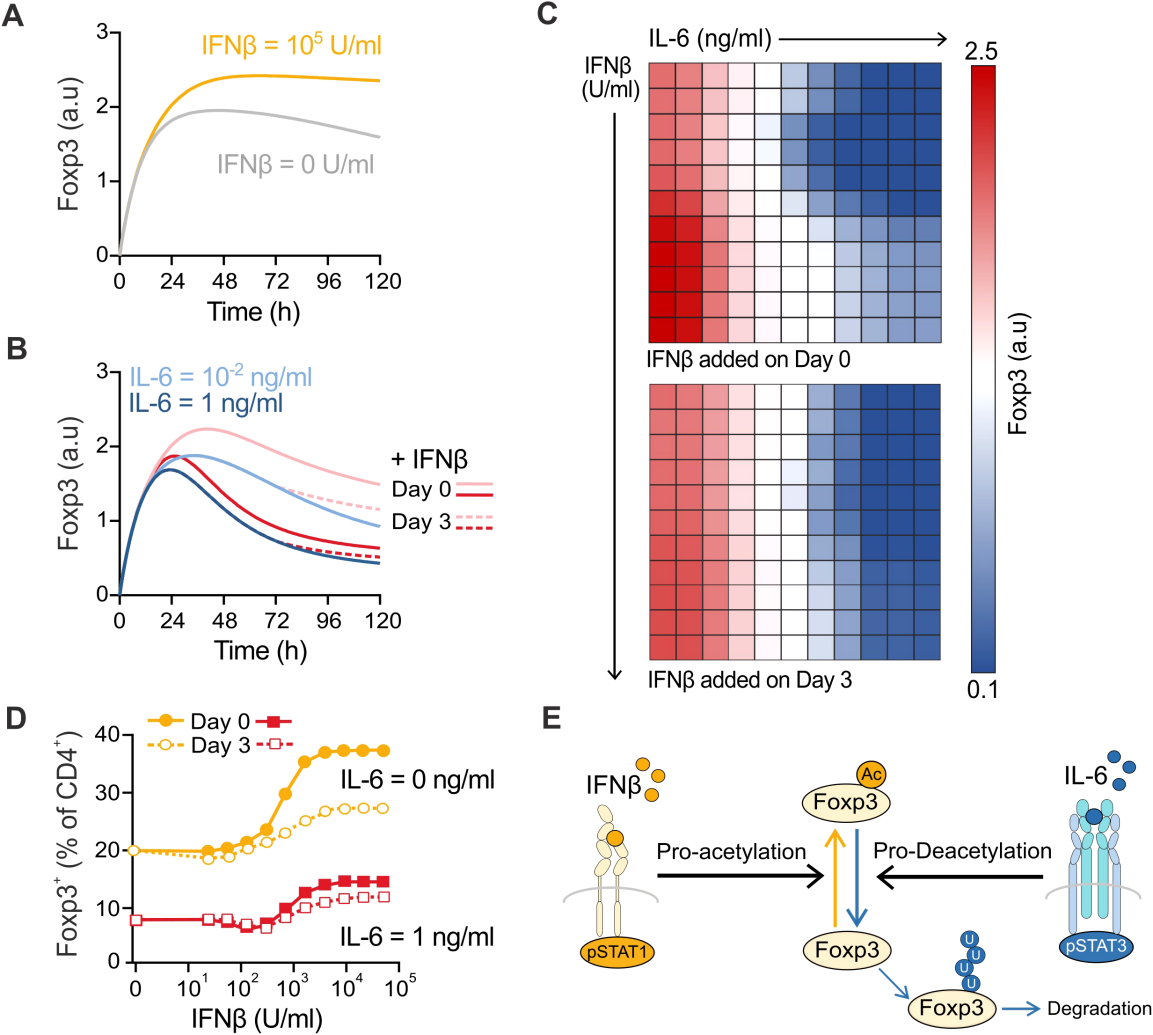
Computational prediction of the molecular interplay of IFNβ and IL-6 in Treg cell induction. **(A)** Computational simulations of the effect of IFNβ (0 to 100,000 U/ml) on the Foxp3 protein levels over 5 days in Treg cell induction cultures. **(B)** Computational simulations of the effect of IFNβ (100,000 U/ml) on the Foxp3 protein levels when added on day 0 (solid line) or day 3 (dotted line) in the presence of IL-6 at 0.01 ng/ml or 1 ng/ml over 5 days in Treg cell induction cultures. **(C)** Heatmaps depicting the combined effect of IL-6 (0 to 20 ng/ml) and IFNβ (0 to 100,000 U/ml) at the end of the simulated culture when IFNβ is added on day 0 (top) or day 3 (bottom). **(D)** Maximum IFNβ effect when added on day 0 (filled squares) or day 3 (empty squares) in the absence (yellow) or presence (red) of IL-6 (1 ng/ml) when the Foxp3 concentration is converted to a predicted percentage of Foxp3^+^ cells in the culture. **(E)** Molecular mechanistic hypothesis guided by the predictions from the computational model.

**Figure 3:**
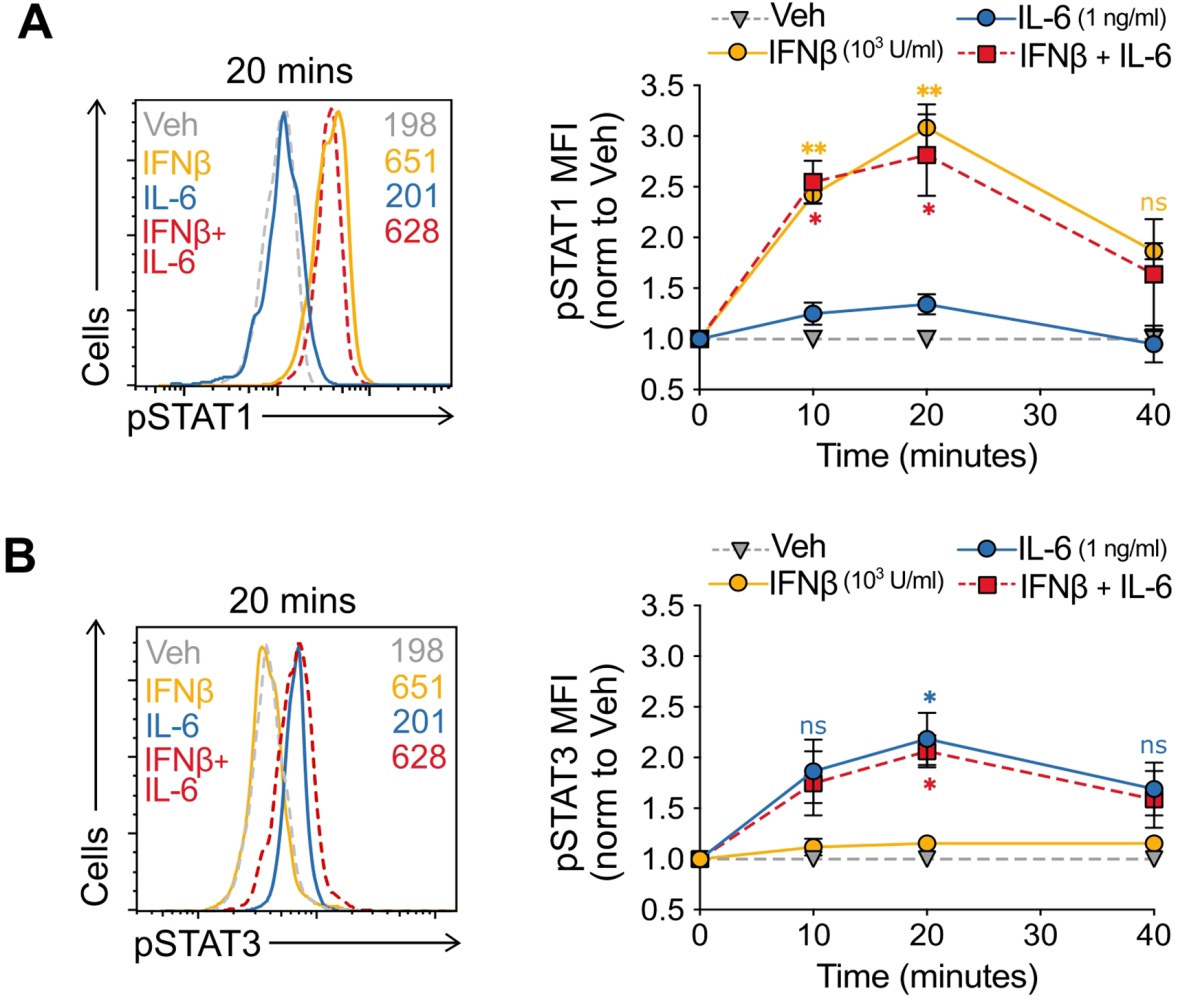
IFNβ does not inhibit IL-6-mediated STAT3 signaling **(A, B)** Evolution over time (right) and representative histogram (left) at peak signaling (20 mins) of pSTAT1 **(A)** and pSTAT3 **(B)** in murine naïve CD4^+^ T cells in the presence of vehicle, IFNβ (1000 U/ml), IL-6 (1 ng/ml), or IFNβ + IL-6 fixed at time points from 0–40 minutes. Summaries depict mean ± SD, n=5 per group, ANOVA with *post hoc* Tukey HSD test, comparing either IFNβ alone **(A)** or IL6 alone **(B)** with IFNβ + IL-6 at each time point; *p<0.05, **p<0.01, ns, not significant.

**Figure 4:**
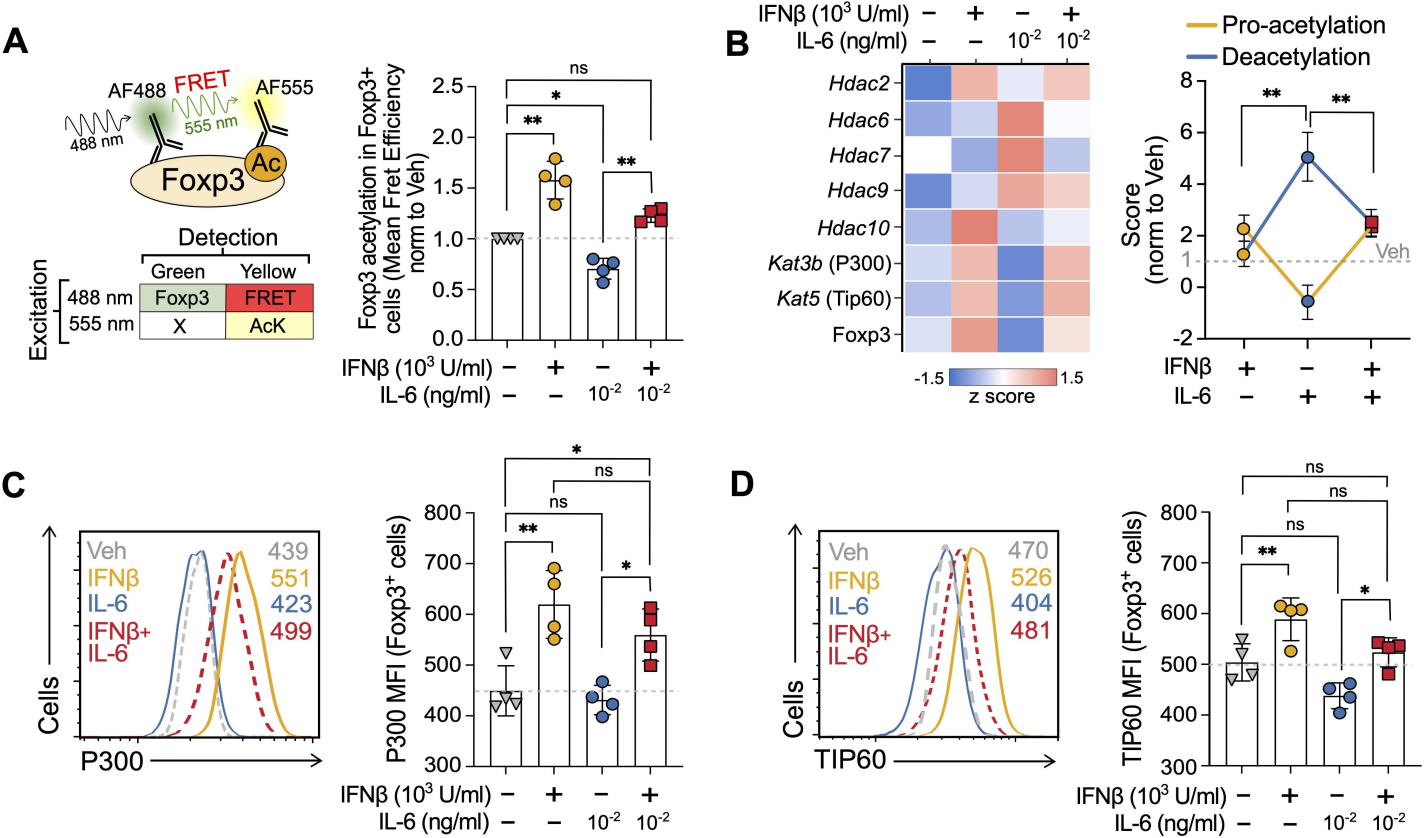
IFNβ prevents IL-6-mediated decrease in Foxp3 acetylation. **(A)** Description of the FRET-based flow cytometry strategy (left) to measure Foxp3 acetylation in Foxp3^+^ cells and results (right) at day 3 of the Treg induction cultures treated with vehicle, IFNβ (1000 U/ml), IL-6 (0.1 ng/ml) or IFNβ + IL-6. **(B)** Heatmap indicating expression of acetyltransferases and deacetylases (left) obtained by transcriptomic analyses (RT-PCR) and combined pro-acetylation and deacetylation score (right) normalized to untreated (see *Methods*) at day 3 of the Treg induction cultures treated with vehicle, IFNβ (1000 U/ml), IL-6 (0.1 ng/ml) or IFNβ + IL-6. **(C, D)** Representative histograms of P300 (encoded by *Kat3b*) and TIP60 (encoded by *Kat5*) in Foxp3^+^ cells at day 3 of the Treg induction cultures treated with vehicle, IFNβ (1000 U/ml), IL-6 (0.1 ng/ml) or IFNβ + IL-6. Summaries depict mean ± SD, ANOVA followed by Tukey *post-hoc* test, *p<0.05, **p<0.01, ns, not significant.

Male and female C57BL/6 were purchased from The Jackson Laboratory (stocks #000664 respectively) and bred at mouse facilities of the Icahn School of Medicine at Mount Sinai. Data depicted in the figures include male and female mice, as we used both for Treg cell inductions (**Figures 1**–**4**).

### Human subjects

**Figure 5:**
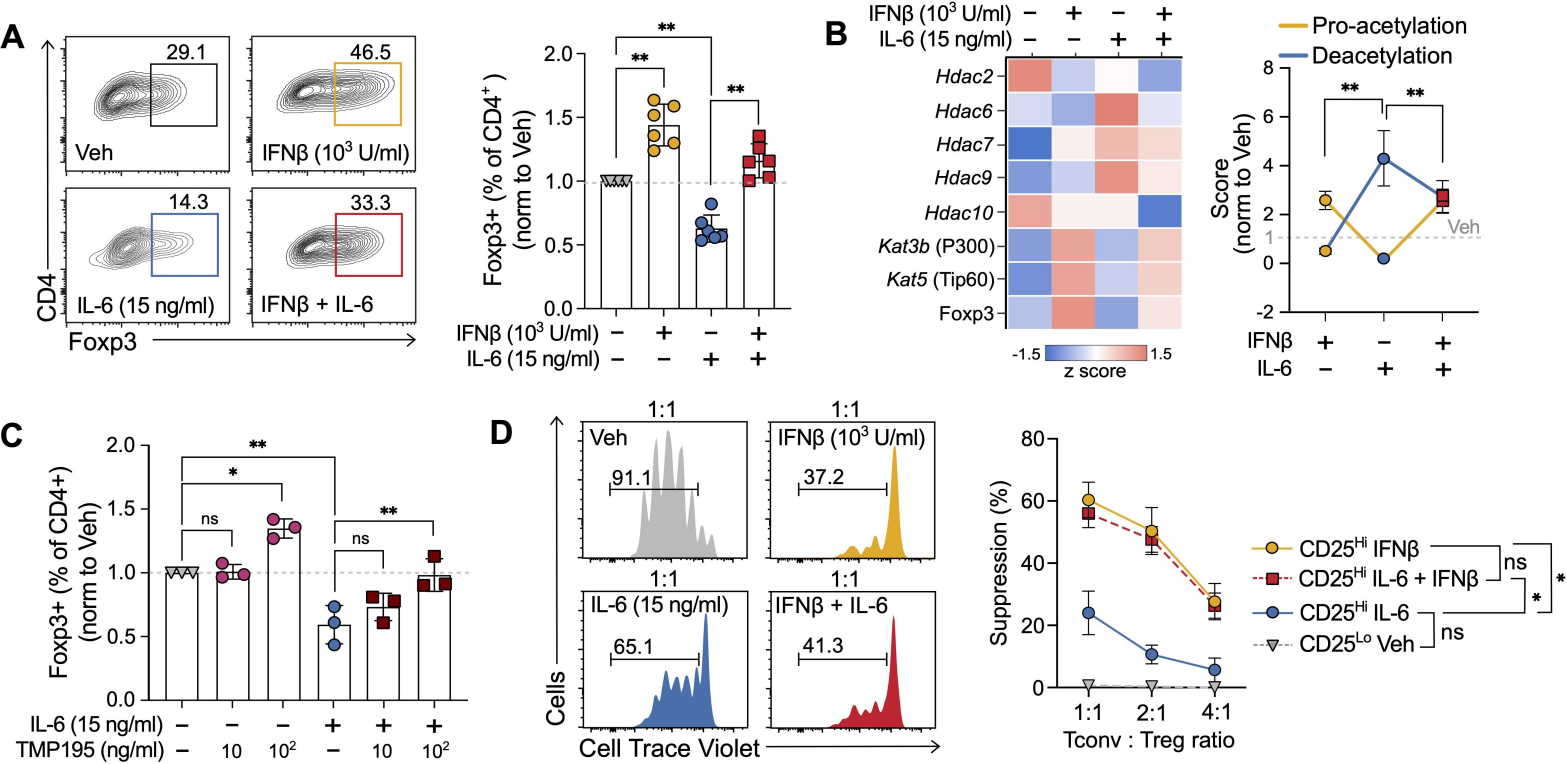
IFNβ counteracts IL-6-mediated effects on human Treg induction. **(A)** Representative scatter plots (left) and summary results (right) of percentage of Foxp3^+^ cells obtained at the end of 5-day human Treg cell induction cultures treated with vehicle, IFNβ (1000 U/ml, day 0), IL-6 (15 ng/ml), or IFNβ + IL-6. **(B)** Heatmap indicating expression of acetyltransferases and deacetylases (left) obtained by transcriptomic analyses (RT-PCR) and combined pro-acetylation and deacetylation score (right) normalized to untreated (see *Methods*) at day 3 of human Treg induction cultures treated with vehicle, IFNβ (1000 U/ml), IL-6 (15 ng/ml) or IFNβ + IL-6. **(C)** Percentage of Foxp3^+^ cells at the end of the 5-day culture in the presence of increasing concentrations of class II HDAC inhibitor TMP195. **(D)** Representative histograms (left) and results summary (right) of the suppression capacity of Treg cells induced in the presence of vehicle, IFNβ (1000 U/ml), IL-6 (15 ng/ml), or IFNβ + IL-6 (n=3). Suppression capacity was calculated as the relative decrease in proliferation of Tconv in the presence of Treg cells. Summaries depict mean ± SD, ANOVA with *post-hoc* Tukey HSD test,*p<0.05, **p<0.01, ns, not significant.

For experiments in human cells (**Figure 5**) we used peripheral blood mononuclear cells (PBMCs) from buffy coats obtained from anonymous donors to the New York Blood Bank.

### CD4^+^ naïve lymphocyte isolation

Spleens were harvested in PBS and mechanically disaggregated through a mesh strainer (70 micrometer (µm)) with the aid of the back of a syringe plunger. PBS was decanted after centrifugation, and red blood cells were lysed (ACK lysis buffer, 2 min at room temperature, Roche). Cells were then centrifuged, re-suspended in PBS, and filtered again through a 70 micrometer (µm) nylon mesh. Human PBMCs were isolated from buffy coats through Ficoll density gradient centrifugation (Histopaque, SigmaAldrich) at 490g. The isolated lymphocyte single‐cell suspension of enriched either spleen or PBMCs leukocytes was processed for different assays or used to isolate naïve CD4^+^ T cells using magnetic separation (EasySep™ Mouse or Human Naïve CD4^+^ T Cell Isolation Kit, STEMCELL Kit) for Treg cell induction cultures.

### Flow cytometry, transcription factor, and acetyltransferase staining

Cells were evaluated for surface antigen expression following incubation with fluorescently conjugated antibodies in PBS or a buffer consisting of 2% rat serum 2 mM EDTA according to the manufacturer’s instructions. For Foxp3, AcK, T-bet, IL-17A, pSTAT1, pSTAT3, P300, and TIP60 staining, cells were permeabilized using an intracellular/transcription factor staining kit (eBiosciences) and stained with Foxp3, AcK, P300, and TIP60 antibodies. Data were acquired on a 3-laser FACSLyric flow cytometer (BD bioscience) and analyzed using FCS Express 7 software.

### *In vitro* Treg cell inductions

Naïve CD4^+^ T cells were enriched from murine splenocytes (CD44^lo^CD62^hi^) or human peripheral blood mononuclear cells (CD45RA^+^CD45RO^-^) (EasySep™ Mouse Naïve CD4^+^ T Cell Isolation Kit, EasySep™ Human Naïve CD4^+^ T Cell Isolation Kit, respectively, STEMCELL Technologies) and their purity checked by flow cytometry. Mouse cultures: 200,000 naïve CD4^+^ T cells were incubated with IL-2 (2.75 ng/ml, Peprotech), TGFβ (2.5 ng/ml), Peprotech) and stimulated with αCD3/αCD28 (15 ml/million cells, Gibco) (polyclonal). Human cultures: 200,000 naïve CD4^+^ T cells were cultured with IL-2 (100 U/ml, BD Pharmingen), TGFβ (3 ng/ml, Peprotech) and stimulated with αCD3/αCD28 (15 ml/million cells, Gibco).

### Treg cell suppression assays

200,000 PBMCs labeled with CellTrace violet were stimulated with αCD3/αCD28 (1ng/ml, BD Biosciences) in the presence of different amounts of polyclonally induced Treg cell as described above, previously sorted as CD4^+^CD25^hi^CD127^-^. The percentage of proliferating cells was determined by flow cytometry based on cell tracker dilution 5 days after stimulation.

### FRET flow cytometry for Foxp3 acetylation

Cells were harvested at the end of 5-day Treg cell induction cultures in the presence or absence of IFNβ or IL-6 and were surface stained with αCD4 antibody together with a fixable viability dye (or an α-Annexin V antibody). For Foxp3, Acetylated lysines (AcK), P300, and TIP60 intracellular staining, cells were fixed/permeabilized using an intracellular/transcription factor staining kit (eBiosciences). Each biological sample was divided into 3 for distinct staining: (i) FRET donor antibody alone (Foxp3) labeled with AF488, (ii) FRET acceptor antibodies alone labeled with AF555 (AcK) and (iii) both together. Antibodies were labeled with the appropriate fluorophores using a protein labeling kit (#A20174, Thermo Fisher Scientific, USA). Samples were analyzed in an Attune NxT flow cytometer (Thermo Fisher Scientific) acquiring either the donor (excitation 488 nm; detection, green; blue1 in Attune) and acceptor channel (excitation 555 nm; detection, yellow; yellow 1 in Attune) alone, or the donor and FRET (excitation 488 nm; detection; blue 4 in Attune) channels together. Additional controls swapping donor and acceptor antibody-proteins were performed. A full list of antibodies used is available in **Supplementary Table S1**.

### FRET flow cytometry analysis

Flow cytometry data was analyzed using FCS express software.

For FRET efficiency calculation flow cytometry.fcs files were parsed using scripts programmed in R. For each cell the apparent FRET efficiency was obtained as:


(1) $$\%FRET\;Eff.=\frac{\left(Acceptor\;Intensity-Mean\;Background\;Acceptor\;Intensity\right)*100}{Donor\;Intensity-Mean\;Background\;Donor\;Intensity}$$

where the Mean Background Acceptor and Donor Intensities were estimated from the counterpart mono-stained samples restricted to CD4^+^ cells. All subtracted intensities that resulted in a negative number were set to 0 to avoid negative FRET efficiencies. A channel with %FRET efficiency with the calculated value for each cell was created in the.fcs file, and the mean %FRET efficiency for each subcompartment (Foxp3^-^ and Foxp3^+^) cells was obtained using conventional gating in FCS Express.

### Quantitative RT-PCR

RNA expression was quantified using quantitative RT-PCR. cDNA was synthesized from total RNA using AffinityScript MultiTemp RT (Agilent) with an oligo(dT)18 primer. Real-time PCR was performed using PlatinumTaq DNA polymerase (Invitrogen) and SYBR Green (Invitrogen) on an ABI7900HT thermal cycler (Applied Biosystems), as described previously (21). A robust global normalization algorithm, using expression levels of the housekeeping genes ribosomal protein S11 (*Rps11*), b-actin (*Actb*), and a-tubulin (*Tuba*), was used for all experiments, as described elsewhere (21, 22). In brief, all crossing threshold values (Ct) were first adjusted by the median difference of all samples from *Actb*. Each individual sample was then further corrected by the median crossing threshold value of the three corrected housekeeping controls for that sample, and the corrected ΔCt was obtained.

### Pro-acetylation and deacetylation transcriptional scores

The pro-acetylation and deacetylation scores were calculated as the sum of the Z-scores of the genes known to have the potential to acetylate (*Kat3b*, *Kat5*) and deacetylate (*Hdac2*, *Hdac6*, *Hdac7*, *Hdac9*, and *Hdac10*) Foxp3. The scores were normalized to untreated to capture the effects elicited by IFNβ, IL-6, or IFNβ + IL-6 (**Figures 4**, **5**), and depicted without normalizing to show the differences between graft-infiltrating lymphocytes and parenchymal cells (**Supplementary Figure S3**).

### Computational model

The details of the CD4^+^ T cell polarization computational model calibrated to the effects of IFNβ on human and murine Treg inductions *in vivo* have been previously published and described (20). To expand the calibration to include the presence of IL-6 in the culture, we simulated the Treg cell-optimized model in the presence of IL-6 at 0.01, 0.1, and 1 ng/ml and in the absence of IFNβ and compared the Foxp3 concentration at day 5 in the model with the experimental results expressed as the percentage of Foxp3^+^ cells from **Figure 1B**. We then performed a linear regression to establish the relationship between the concentration of Foxp3 in the model and the percentage of Foxp3^+^. Details and curves are provided in **Supplementary Data Sheet S1**.

### Code and computational model availability

All the R scripts to derive the pro-acetylation and deacetylation scores, as well as the computational model implemented in COPASI are available in GitLab (https://gitlab.com/miguel_fribourg/treg_and_ifnb), including instances of the model that scan the IFNβ and IL-6 combinations used to generate the panels in **Figure 2**.

## Results

### IFNβ prevents the inhibition of Treg cell induction by IL-6

Given that IFNβ and IL-6 have been described to have opposite effects on Treg cell induction (18, 20), we first decided to test whether IFNβ could counteract the inhibitory effects of IL-6 on murine Treg cell induction cultures (**Figure 1A**). To set up these cultures, we isolated naïve splenic CD4^+^ T cells (defined as CD44^lo^ CD62^hi^ purity >95%) from wild-type C57BL/6 (B6) animals, cultured them for 5 days with αCD3/αCD28 activating beads under Treg cell polarizing conditions (IL-2 and TGFβ) in the presence of either vehicle or increasing concentrations of IL-6 with or without IFNβ. At the end of the culture, we used flow cytometry to determine the frequency of Foxp3^+^ cells (Treg).

As previously reported, the addition of IFNβ at 1,000 U/ml, a concentration shown to maximize the Treg cell-enhancing effect (20), significantly increased the frequency of Foxp3^+^ cells (from 19.46 ± 0.52% to 37.48 ± 3.68%) (**Figure 1B**). IL-6 alone inhibited Treg cell induction in a dose-dependent manner even at markedly low concentrations (0.01 ng/ml), with this effect not being attributable to a decrease in the viability of already formed Treg cells, or reduction of absolute cell number counts in the culture (**Supplementary Figure S1**). Of note, IFNβ does not polarize cells to Treg in the absence of IL-2 and TGF-β (20). Consistent with our hypothesis, IFNβ counteracted the inhibitory effects of IL-6, significantly limiting the reduction in the percentage of Foxp3^+^ cells at the end of the Treg cell induction cultures for all IL-6 concentrations. Similarly, increasing the concentration of IFNβ from 10 to 10,000 U/ml was able to rescue the maximum Treg inhibitory effect obtained with 0.1 ng/ml of IL-6 (**Figure 1B**). We used the surface marker neuropilin 1 (Nrp1) (23, 24) to further confirm that the observed effects pertained to Treg peripherally induced from CD4^+^ naïve T cells (Foxp3^+^Nrp1^-^) and not to thymic-derived natural Treg (nTreg, Foxp3^+^Nrp1^+^). Of note, in all groups Treg showed similar protein expression of transcription factor Helios, associated, which has been associated with Treg stability (25, 26) (**Supplementary Figure S2**).

Inflammatory cytokines have been shown to polarize naïve T cells to an effector phenotype (19, 27). In particular, IFNβ can enhance Th1 cell polarizations (28), and the combination of IL-6 with TGFβ is canonical to polarize cells to IL-17-producing Th17 cells (29, 30). As a control, we tested whether the differences in Treg cell frequencies observed in the culture could be explained by changes in the percentage of Th1 cells (T-bet^+^) and/or Th17 cells (IL-17A^+^). The frequencies of Th1 cells and Th17 cells remained below 2% for all treatments (**Figure 1C**).

To determine whether IFNβ prevents IL-6-mediated Treg cell inhibition or rescues the cells, in an analogous experiment, we added IFNβ to the cultures on day 3 and compared the effect to that of IFNβ addition at the beginning of the culture (day 0). Interestingly, delayed treatment with IFNβ following IL-6 did not rescue the decrease in Foxp3^+^ cells at the end of the culture (**Figure 1D**). Together, these results support the concept that IL-6 and IFNβ exert opposing effects on Treg cell induction, and IFNβ can prevent but not revert its inhibition by IL-6.

### Computational prediction of the molecular interplay between IFNβ and IL-6

To investigate the molecular mechanism underpinning IFNβ’s ability to prevent IL-6-mediated Treg, we interrogated a mechanistic Ordinary Differential Equation (ODE) computational model of CD4^+^ polarization. This computational model constitutes an expansion of a publicly available (https://www.ebi.ac.uk/biomodels/BIOMD0000000451) ODE model developed at the Virginia Bioinformatics Institute (31), which incorporates interleukin-6 (IL-6) and its signaling, and was later expanded by our group to include type I interferon signaling. It includes 60 differential equations representing 52 reactions and 93 species, and is implemented and simulated in COPASI (32), an open-source software application used for simulating and analyzing biochemical networks and systems biology model, fully compatible with Systems Biology Markup Language (SBML). Full details on model calibration can be found in **Supplementary Data Sheet S1** and (31).

*Model assumptions*. The most relevant assumptions for the use of the model for this work are the following:

- The model assumes correct engagement of the T-cell receptor (TCR) (signal 1) and co-stimulatory receptors (signal 2). In that sense, the model is designed to explore the effects of different cytokine inputs (signal 3) on T cells.
- The model does not explicitly describe/include T cell proliferation, but rather describes the system as one stereotypical cell with varying concentrations of species, Tbet, GATA-3, RORγt, and Foxp3, which can successfully be mapped to the frequencies of different subsets T cell subsets (Th1, Th2, Th17, and Treg).

This model has been calibrated to data from the literature and to our own experimental data, and incorporates the optimized phospho-STAT signaling pattern (pSTAT1, pSTAT3, pSTAT4, pSTAT5, pSTAT6) elicited by IFNβ to mediate its Treg cell-enhancing effects (see **Supplementary Data Sheet S1** section for details on how the optimized signaling pattern and calibrations to IFNβ and IL-6 concentrations were derived).

We first simulated the interplay between a wide range of IFNβ and IL-6 concentrations and study its effects on Treg induction by monitoring Foxp3 concentration in the cell over time. As in our cell culture experiments, we compared the effect of adding IFNβ on day 0 with IL-6, or 3 days post-induction. In our simulations, the presence of IL-6 also reduces the amount of Foxp3 at the end of the culture in a dose-dependent fashion, indicating the adequate calibration of the model (**Figures 2A, B**). In the simulations, IFNβ decelerated the IL-6-mediated Foxp3 decrease, but its addition on day 3 does not afford it enough time to counteract this effect by day 5 (**Figure 2B**). Looking at the combined impact on Foxp3 concentration at the end of the culture for a wide range of IL-6 and IFNβ concentrations added at day 0, our simulations recapitulated the generalized Treg protective effect of IFNβ observed experimentally. Similarly, the maximum IFNβ protective effect was markedly diminished in the simulations when IFNβ was added on day 3, resulting in approximately half of the percentage of Foxp3^+^ cells at the end of the culture simulations (54.7% reduction on average for all IL-6 concentrations) (**Figures 2C, D**).

The computational model allows us to investigate the predicted molecular mechanisms involved in this Treg protective effect. When looking into the molecular species responsible for the concentration dynamics of Foxp3, we identified that a differential effect of pSTAT1 and pSTAT3 signaling on the reaction rate that controls the conversion of Foxp3 to acetylated Foxp3 in the model governed the simulation results. Thus, the model puts forward the mechanistic hypothesis that IFNβ and IL-6 signal independently through pSTAT1 and pSTAT3, respectively, eliciting opposing effects on the acetylation/deacetylation balance on the Foxp3 protein in CD4^+^ naïve T cells and thereby controlling the extent of Treg formation (**Figure 2E**).

### IFNβ does not alter IL-6-mediated STAT3 signaling

Both IL-6 and IFNβ ligate JAK-STAT coupled receptors and can activate different STATs depending on context and cell type. In most immune cells IL-6 predominantly signals through pSTAT3 (33), while IFNβ does it through pSTAT1 (34, 35). To experimentally address the computational mechanistic hypothesis, we first asked whether IL-6 and IFNβ could alter each other’s STAT signaling. We set up identical parallel Treg induction cultures in the presence of vehicle, IFNβ alone, IL-6 alone, and IFNβ + IL-6. We fixed the cells 0, 10, 20, and 40 minutes following activation and captured the changes in signaling through simultaneous pSTAT1 and pSTAT3 staining and flow cytometry.

Phospho-STAT activation upon IFNAR ligation is typically transient, increasing within minutes and being shut down through clathrin-mediated endocytosis of the activated receptor, and lysosomal degradation (36). Upon IFNβ stimulation, we observed a rapid increase in pSTAT1 activation that peaked at 20 min (3-fold increase) and had already decreased to 1.5-fold by 40 min. Addition of IL-6 only marginally elicited pSTAT1 increase, and more importantly, did not alter the peak levels and the kinetics of IFNβ-mediated pSTAT1 signal. (**Figure 3A**).

Transient IL-6 pSTAT3 activation is associated with a pro-inflammatory transcriptional program in immune cells (37, 38). Our experiments captured this transient dynamic with a peak at pSTAT3 at 20 min post-stimulation with IL-6 alone (2-fold increase). Similarly, addition of IFNβ did neither elicit a pSTAT3 signal, nor modified the IL-6-mediated pSTAT3 activation in these cells (**Figure 3B**).

Taken together, and consistent with the mechanistic model prediction, our results indicate that IFNβ + IL-6 elicited the same extent and temporal dynamics of pSTAT1 and pSTAT3 activation as IFNβ and IL-6 alone, respectively.

### IFNβ prevents IL-6-mediated decrease in Foxp3 acetylation

Guided by the computational model, we next tested the predicted opposing effects of IFNβ and IL-6 on Foxp3 acetylation. To measure Foxp3 acetylation, we used a versatile FRET-based method compatible with flow cytometry developed in the lab (39). This method, previously validated against proximity ligation assays and western blots, allows us to selectively monitor the extent of Foxp3 acetylation in the Foxp3+ population (**Equation 1**) at the end of the cultures (**Figure 4A**, left). As previously reported by our group, IFNβ increased by 58% the acetylation of Foxp3 selectively in the Foxp3^+^ population, while IL-6 alone (0.1 ng/ml) reduced it by 30% compared to vehicle. Interestingly, and consistent with our hypothesis, the addition of IFNβ at the beginning of the culture prevented the IL-6-mediated Foxp3 deacetylation and even increased it by 22% over vehicle controls, suggesting competing effects of both processes on Foxp3 acetylation (**Figure 4A**, right).

To delve into the mechanisms underlying these differences in Foxp3 acetylation, we looked at the changes in the transcription of the genes encoding for the acetyltransferases (KAT; P300 encoded by *Kat3b* and TIP60 encoded by *Kat6*) and deacetylases (HDAC; *Hdac2*, *Hdac6*, *Hdac7*, *Hdac9*, and *Hdac10*) described to mediate Foxp3 acetylation and deacetylation, respectively (39–42). To assess the overall effect on Foxp3, we summarized their change as a pro-acetylation and deacetylation score using as a reference the untreated cells (see *Materials and Methods*) (**Figure 4B**). Compared to control, IFNβ significantly increased the pro-acetylation score through *Kat3b* and *Kat5*, while also modestly increasing deacetylation, primarily driven by *Hdac2* and *Hdac10.* Of note, multiple studies indicate that KATs and HDACs modulate each other expressions and activities to maintain the KAT/HDAC balance (43, 44). In contrast, IL-6 dramatically shifted this balance towards deacetylation by suppressing the pro-acetylation genes and *Hdac6*, *Hdac7*, and *Hdac9*. Paralleling our previous Foxp3 acetylation results (**Figure 4A**), adding IFNβ to IL-6 prevented the decrease of the pro-acetylation and the increase of the deacetylation scores preserving a gene transcription balance that favors Foxp3 acetylation (**Figure 4B**). The observed gene expression differences for acetyltransferases P300 (*Kat3b*) and TIP60 (*Kat5*) were confirmed in terms of protein expression in Foxp3^+^ cells (**Figures 4C, D**).

These results experimentally support the computational mechanistic hypothesis of opposing effects of IL-6 and IFNβ on Foxp3 acetylation in these cells.

### IFNβ counteracts IL-6-mediated effects on human Treg induction

To understand the relevance of these results to human cells, we performed analogous 5-day Treg cell induction experiments using naïve CD4^+^ T cells (CD45RA^+^CD45RO^−^, >95% purity assessed by flow cytometry) magnetically enriched from anonymous donor peripheral blood mononuclear cells (PBMCs). As in our murine experiments, IFNβ significantly increased the percentage of Foxp3^+^ cells at the end of the culture. While the addition of IL-6 inhibited Treg cell induction by approximately 50%, IFNβ was able to prevent this reduction (**Figure 5A**, compare with **Figure 1B**).

HDAC7 and HDAC9, two class II histone deacetylases, have been implicated explicitly in Foxp3 deacetylation in human cells (40, 45). Indeed, in our cultures, the class II HDAC inhibitor TMP195 prevented the inhibitory effects of IL-6 on Treg cell inductions in a dose-dependent fashion (**Figure 5B**). Consistently, transcriptional analysis of the effects of IL-6, IFNβ, and IL-6 + IFNβ revealed that the IL-6 mediated Treg cell induction inhibition was associated with an increase in the deacetylation score driven by *Hdac6*, *Hdac7*, and *Hdac9*, and a concomitant decrease of the pro-acetylation score. As in murine cultures, the transcriptomic change in acetylation balance on Foxp3 was prevented by IFNβ (**Figure 5C**).

IL-6 has been reported not only to hinder Treg induction but also to reduce Treg function (26). To test whether IFNβ could also prevent the IL-6-mediated loss of suppressive capacity, we performed suppression assays. At the end of our Treg cultures, we sorted CD4^+^CD125^hi^CD127^-^ cells and assessed their ability to suppress the proliferation of conventional T (Tconv) cells labeled with a proliferation dye and stimulated by αCD3/αCD28. These assays demonstrated that although Treg induced in the presence of IL-6 exhibited a markedly reduced suppressive capacity when compared with those induced with IFNβ, the presence of IFNβ in the culture was able to prevent this loss in Treg function (**Figure 5D**).

## Discussion

We used a synergistic computational/experimental approach in which the experiments inform the computational model, and the computational model refines the experiments. This allowed us to explore the effect of a large number of combinations of IL-6 and IFNβ in minutes, which would not have been feasible experimentally, and extract the most informative combinations to generate mechanistic hypotheses, thus highlighting the utility of this approach to study cytokine interplay and accelerate research. The core finding of the present work is that IFNβ can prevent IL-6-mediated inhibition through a mechanism that promotes Foxp3 acetylation and opposes the IL-6 deacetylating effects on this Treg master transcription factor regulator. In contrast, IFNβ fails to rescue IL-6 mediated Treg inhibition, which could be explained as a result of IL-6’s head start in this process. Given that many of the acetyltransferases and deacetylases that acetylate Foxp3 also have acetylase on histones (45–47), the lack of rescue might be due at least in part to epigenomic changes, particularly those that might impact the regulation of the gene encoding for Foxp3. Similarly, metabolic effects on T cells triggered by IFNβ and IL-6 might also contribute to our observations, in particular those involved in the synthesis and degradation of acetyl coenzyme A, a crucial carrier for acetyl groups in the cells.

Our findings could be relevant for contexts where IL-6 and IFNβ occur simultaneously and drive T cell responses, such as autoimmune disease. The inflammatory pathogenic role of IL-6 is recognized in many autoimmune diseases, including rheumatoid arthritis (RA) and systemic lupus erythematosus (SLE), and thus IL-6 signaling is targeted therapeutically in many of them (3, 48, 49). Furthermore, the interplay between IFNβ and IL-6 is likely to be affected in the context of the use of jakinibs, small-molecule therapeutics for autoimmune disease that inhibit the activity of one or more of the Janus kinase (JAK) enzymes to shape the STAT signaling in immune cells (50, 51). Interestingly, type I interferons are also believed to play a pathogenic role in SLE, but type I interferon receptor blockade with anifrolumab has yielded mixed results in clinical trials, with benefits being restricted to secondary endpoints (e.g., reduction in glucocorticoid use, or annual flare rate) only in a subset of patients (52). Conversely, IFNβ has been used for three decades to treat remitting-relapsing multiple sclerosis (53) to reduce flare-ups and inflammation, further highlighting the need to better understand the interplay between these two cytokines in order to guide clinical intervention.

Organ transplantation often leads to ischemia-reperfusion injury (IRI), an inflammatory response triggered by tissue damage and oxidative stress. Mechanistic evidence indicates that early post-transplant IRI elicits persistent inflammatory cytokines, including IL-6 and IFNβ, which promote donor-reactive effector (Teff) and memory (Tmem) (54–58). Building upon the success of anti-IL-6R therapy in RA (Tocilizumab, TCZ) (59), TCZ is currently being explored in the transplant recipient population (55, 60, 61). In a recently published mechanistic study in a murine model of transplant, Muckenhuber et al. showed that TCZ could increase Treg and reduce Teff in the graft (62). To validate the molecular mechanism put forward by our study, we calculated the pro-acetylation and de-acetylation scores as in **Figure 4** in the publicly available gene expression data (RNA-seq) obtained from the graft cells in this study. While TCZ increased the pro-acetylation and decreased the de-acetylation Foxp3 scores in graft infiltrating lymphocytes when compared with untreated, the scores remained stable with and without TCZ in the parenchymal cells (**Supplementary Figure S3**), further supporting the *in vivo* relevance of our *in vitro* studies.

Our study has limitations. Although the effect of IL-6 on Treg induction and suppressive capacity of peripherally induced Treg has been well described (26), further studies are required to clarify the impact and mechanisms through which IL-6 and IFNβ signaling regulate Foxp3 expression, stability, and suppressive capacity in thymic natural Treg. Thus, our findings regarding the interplay of these cytokines are likely only pertinent to peripheral tolerance. We also acknowledge that our focus has been limited to IFNβ and that other type I interferons might have different effects. Finally, the temporal dynamics of IL-6 and IFNβ differ based on context and disease. For example, we did not specifically explore how IL-6 would affect Treg after they are induced in the presence of IFNβ.

The present work has important pharmacological implications. The role of specific histone deacetylases (HDACs) in T cell function at large and Foxp3 acetylation in particular has been established in the field (40, 41, 63). The design and identification of selective HDAC inhibitors has been notoriously challenging (64), as HDACs are extraordinarily promiscuous, making balancing the acetylation flux in the cells complicated. Our finding that IFNβ can maintain the acetylation environment is of therapeutic relevance, as it has FDA approval, and provides a deeper understanding of its effectiveness in certain contexts, which may pave the way for new therapeutic strategies.

## Conclusions

The signaling interplay between cytokines and their combined effects in different contexts and cell types remains a challenge in the immunology field, exacerbated by the fact that most cytokines have pleiotropic effects. Here, we demonstrate that IFNβ, an antiviral pro-inflammatory cytokine, can protect from the hindering effects of IL-6 on Treg induction and suppressive capacity. Our results, guided by a computational mechanistic model, reveal that molecularly, IL-6 and IFNβ signal independently to promote a pro-acetylation and deacetylation environment, respectively, exerting opposing effects on Foxp3 acetylation, the master transcription factor regulator in Treg. We also demonstrate that this mechanism is present in murine and human cells, further enhancing the relevance of these results.

## Data Availability

Existing datasets are available in a publicly accessible repository: Publicly available datasets were analyzed in this study. GEO accession: GSE241471.

## Supplementary material

Supplementary Data Sheet 1 Computational Model Details.

Supplementary Table 1 Reagents.
