# Supplementary material for: Interferon-β and interleukin-6 exert opposing effects on Foxp3 acetylation to control regulatory T cell induction
Source: Front Immunol. 2025 May 30;16:1593931. doi: 10.3389/fimmu.2025.1593931 (PMC12162494; doi:10.3389/fimmu.2025.1593931)
Supplement: Supplementary Data Sheet 1 — Computational Model Details. [file DataSheet1.pdf]

Supplementary Materials

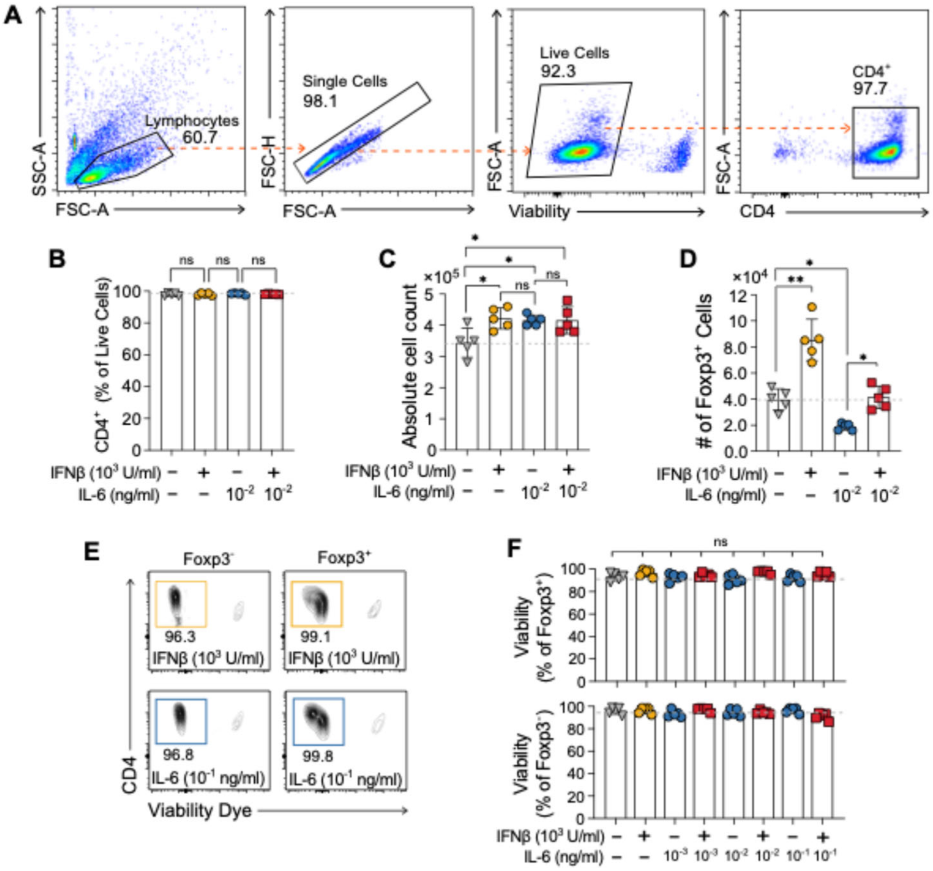

Figure S1. Effects of IL-6 on cell viability in Treg induction cultures. (A) Gating strategy for murine Treg induction cultures. **(B-C)** Summaries of percentage of CD4+ T cells (B), Absolute cell count (C) and Number of Foxp3+ cells (D) at the end of 5-day murine Treg induction cultures treated with vehicle, IFNβ (1000 U/ml), IL-6 (0.1 ng/ml) or IFNβ + IL-6. **(E)** Representative scatter plots (left) and summary of the percentage of live Foxp3+ and Foxp3- cells at the end of 5-day murine Treg induction cultures treated with vehicle, IFNβ (1000 U/ml), IL-6 (0.01, 0.1, 1 ng/ml) or IFNβ + IL-6. Summaries depict mean ± SD, n=5 per group, ANOVA with post hoc Tukey HSD test, *p<0.05, **p<0.01, ns, not significant).

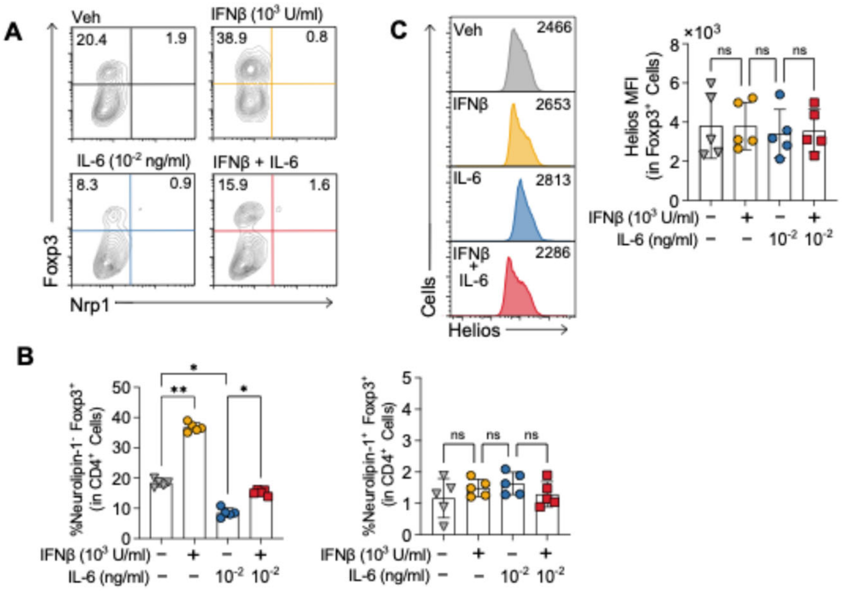

Figure S2. Flow cytometric analysis of Treg cell markers in murine Treg induction cultures. (A-B) Representative scatter plots (A) and summary of the percentage of Nrp1$^+$Foxp3$^+$ and Nrp1$^-$ Foxp3$^+$ cells at the end of 5-day murine Treg induction cultures treated with vehicle, IFNβ (1000 U/ml), IL-6 (0.1 ng/ml) or IFNβ + IL-6. **(C)** Representative histogram (left) and summary of the mean fluorescence intensity of transcription factor Helios in Foxp3$^+$ cells at the end of 5-day murine Treg induction cultures treated with vehicle, IFNβ (1000 U/ml), IL-6 (0.1 ng/ml) or IFNβ + IL-6. Summaries depict mean ± SD, n=5 per group, ANOVA with post hoc Tukey HSD test, *p<0.05, **p<0.01, ns, not significant).

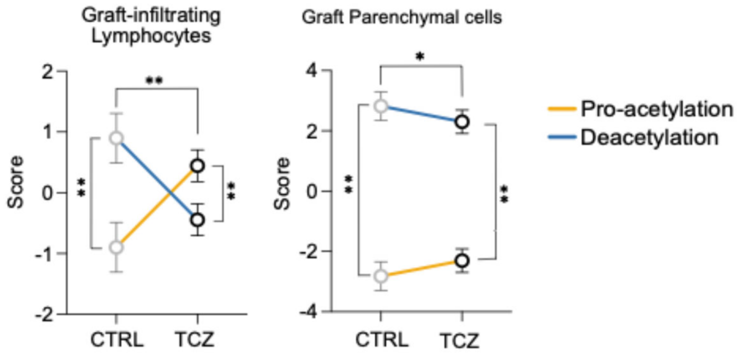

Figure S3. Pro-acetylation and acetylation gene scores in murine transplant recipients with TCZ. Combined pro-acetylation and deacetylation score normalized to untreated (see *Materials and Methods*) calculated from publicly available transcriptomic data of graft-infiltrating lymphocytes and graft parenchymal of BALB/c heart C57BL/6 transplant recipients cells treated in the absence (CTRL) or presence of tocilizumab (TCZ). Details of the transplants and treatments are provided in Muckenhuber et al. 2024 (mean ± SEM, n=4 per group, ANOVA with post hoc Tukey HSD test, * p<0.5, ** p<0.01).

Supplementary Data S1 | Computational Model Details, refers to Figure 2

1. Model Creation

The computational model used for this work constitutes an expansion of a publicly available (https://www.ebi.ac.uk/biomodels/BIOMD0000000451) ordinary differential equation (ODE) model developed at the Virginia Bioinformatics Institute (1), which already included interleukin-6 (IL-6) and its impact on T cell polarization, and was later expanded by our group to include type I interferon signaling (2). The model includes 60 differential equations representing 52 reactions and 93 species, and is implemented and simulated in COPASI (3), an open-source software application used for simulating and analyzing biochemical networks and systems biology model, fully compatible with Systems Biology Markup Language (SBML). Full details on model calibration can be found in (1).

Model assumptions. The most relevant assumptions for the use of the model in this work are the following:

- The model assumes correct engagement of the T-cell receptor (TCR) (signal 1) and co-stimulatory receptors (signal 2). In that sense, the model is designed to explore the effects of different cytokine inputs (signal 3) on T cells.
- The model does not explicit describe/include T cell proliferation, but rather describes the system as one stereotypical cell with varying concentrations of species, Tbet, GATA-3, RORγt, and Foxp3, which can successfully be mapped to the frequencies of different subsets T cell subsets (TH1, TH2, TH17, and TREG).

Model expansion strategy. In order to expand the model to include the effects of interferon-beta (IFNβ) on T cell polarization we introduced two additional species: the concentration of IFNAR (type I interferon receptor in the membrane), and the concentration of IFNβ as one of the cytokines in the environment as a new input to the cell. The implementation of the IL-6 receptor signaling and its coupling to STAT3 is analogous to the one described below for IFNAR.

It has been shown that type I interferons can signal through phosphorylation of different STATs depending on cell type and context (4, 5). In order to make the model flexible, we allowed the ligated type I interferon receptor (IFNAR/IFNβ) to phosphorylate STAT1, STAT3, STAT4, STAT5, and STAT6 through the control of 5 coupling constants, $\alpha 1, \alpha 3, \alpha 4, \alpha 5$, and $\alpha 6$, respectively: This results in the addition to the model of:

1. An ODE that describes the binding of the IFNβ in the environment to the IFNAR receptor, $IFN\beta + IFNAR1 \leftrightarrow IFN\beta/IFNAR1$ as a reversible mass action equation combined with a Hill equation that depends on the concentration of suppression of cytokine signaling 1 (SOCS1) bound to regulate Janus kinases (JAKS), which are responsible for STAT signaling

$$\frac{d[IFN\beta/IFNAR1]}{dt} = Vf[IFN\beta][IFNAR1]\frac{K^2}{[SOCS1/JAKS]^2 + K^2} - Vr[IFN\beta/IFNAR1], \quad (1)$$

where all concentrations vary over time and the parameters *Vf*=0.1, *K*=0.2639, and *Vr*=0.1 were chosen based on the parameters used to describe cytokine binding to similar receptors already in the model, such as the IL-6 or the IL-2 receptors.

2. An activation term in the ODEs that govern STAT1, STAT3, STAT4, STAT5, and STAT6 phosphorylation, $STAT_i \leftrightarrow STAT\_P_i$. These ODEs are in the model of the form:

$$\frac{d[STAT\_P_i]}{dt} = Vf[STAT_i] \cdot I \cdot A - Vr[STAT_{P_i}], \quad (2)$$

where all concentrations vary over time. The parameter *I* includes all the inactivation terms mediated by the different species and has the general form of

$$I = \prod_{i=1}^{ni} \frac{KI_i^2}{[I_i](t)^2 + KI_i^2}, \quad (3)$$

and *A* includes all the activation terms mediated by the different species of the general form

$$A = \sum_{i=1}^{na} \frac{[A_i](t)^2}{[A_i](t)^2 + KA_i^2}, \quad (4)$$

being $[I_i]$ and $[A_i]$ the concentrations of the inhibitory and activating species as a function of time, respectively, and KI_i and KA_i are specific constant for each species that modulate their effects.

To include the coupling of IFNβ-mediated signaling to each of the STATs, we added an activation term preceded by a coupling parameter α as:

$$\alpha \frac{[IFNAR1/IFN\beta]^2}{[IFNAR1/IFN\beta]^2 + K^2}. \quad (5)$$

We also included the following changes in four of the parameters from the original model to ensure the proper behavior of the model (see section 2) in the presence of the new receptor.

- in equation re15 (Tbet↔TbetP): K3 change from 3.58849 to 1e-3
- in equation re13 (STAT1↔STAT1P): K1 change from 5.04432 to 1
- in equation re23 (GATA3↔GATA3P): K4 from 0.321065 to 1e-5 and K5 from 0.1 to 1e-5

2. Optimization of IFNβ effects on T_H1, T_H2, T_H17 and T_{REG} polarizations

We verified that, in the absence of IFNβ, the expanded model adequately polarized $CD4^+$ naïve T cells to T_H1 (in the presence of IL-12, IL-18 and IFNγ), T_H2 (in the presence of IL-4 and IL-2), T_H17 (in the presence of IL-6 and TGF-β) and T_{REG} (in the presence of IL-2 and TGF-β). We tested *in silico* the potential effects of IFNβ on each of these polarizations by allowing the coupling (weights) of IFNAR to the different STATs to vary in the model such that they maximized the effect of IFNβ on each of the polarizations. To achieve this we found the vector of STAT-coupling weights that maximized the overall concentration of the active form of the appropriate transcription factor (Tbet for T_H1, GATA-3 for T_H2, RORγt for Th17, and Foxp3 for T_{REG}) with respect to the others in the span of 5 days (120 hour). We implemented these searches as an optimization problem in which we maximized the corresponding cost functions

$$fT_H1 = \sum_{ti=1}^{120} |[Tbet](ti)^2 - [GATA3](ti)^2 - [ROR\gamma t](ti)^2 - [Foxp3](ti)^2| \quad (6)$$

$$fT_H2 = \sum_{ti=1}^{120} |[GATA3](ti)^2 - [Tbet](ti)^2 - [ROR\gamma t](ti)^2 - [Foxp3](ti)^2| \quad (7)$$

$$fT_H17 = \sum_{ti=1}^{120} |[ROR\gamma t](ti)^2 - [Tbet](ti)^2 - [GATA3](ti)^2 - [Foxp3](ti)^2| \quad (8)$$

$$fT_{REG} = \sum_{ti=1}^{120} |[Foxp3](ti)^2 - [Tbet](ti)^2 - [GATA3](ti)^2 - [ROR\gamma t](ti)^2| \quad (9)$$

for the parameter vector $\vec{\alpha} = [\alpha 1, \alpha 3, \alpha 4, \alpha 5, \alpha 6]$ with values constrained to $\alpha i \in [0,1]$ and initial values=0.5. We solved these highly non-linear problems using a random search algorithm implemented in COPASI with 1000 iterations (in average ~500 iterations were enough to reach convergence). Optimization was run 10 times for each polarization and the optimized models for each polarization parametrized with the average values for α1, α3, α4, α5, and α6 of the 10 runs (see **Table 1**).

	Average					SD				
	α1	α3	α4	α5	α6	α1	α3	α4	α5	α6
T_H1	0.904	0.201	0.967	0.987	0.249	0.067	0.045	0.0526	0.032	0.002
T_H2	0.01	0.39	0.19	0.87	0.28	0.0023	0.025	0.0067	0.0087	0.00065
T_H17	0.105	0.912	0.166	0.097	0.307	0.045	0.0263	0.022	0.067	0.0087
T_{REG}	0.983	0.001	0.605	0.858	0.098	0.0013	0.0002	0.023	0.078	0.0012

Table 1. **Optimized weights for each polarization**. Average and standard deviation (SD) of 10 optimization runs.

We first validated that the model that includes the T_{REG} effect recapitulated the biology of T cell polarization. We ran computational simulations of the differentiation from the naïve state into T_H1 (IL-12 + IL-18 + IFNγ) T_H2 (IL-2 + IL-4), T_H17 (IL-6 + TGFβ) and T_{REG} (IL-2 + TGFβ) (**Figure 1**)

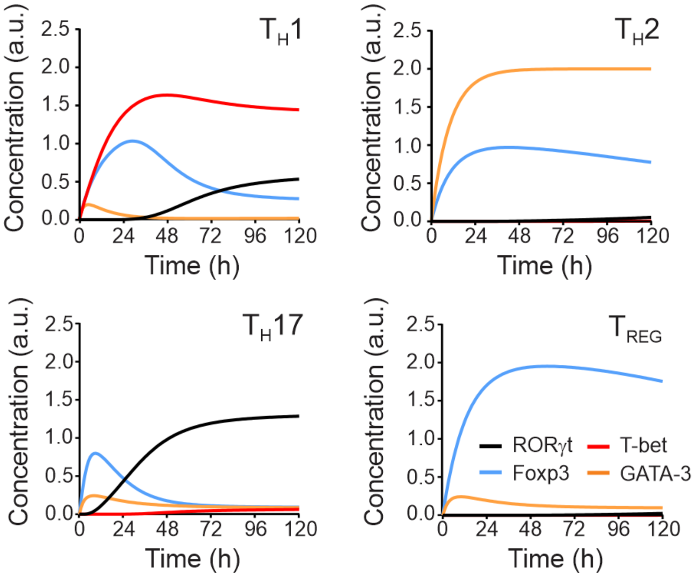

Figure 1. Simulations of the optimized model in T_H1, T_H2, T_H17 and T_{REG} polarizing conditions.

We then simulated the optimized model for each polarization at IFNβ concentrations ranging from 0 to 10,000 U/ml. Simulations indicate a greater and unexpected proportional effect of IFNβ on the T_{REG} polarization (**Figure 2**).

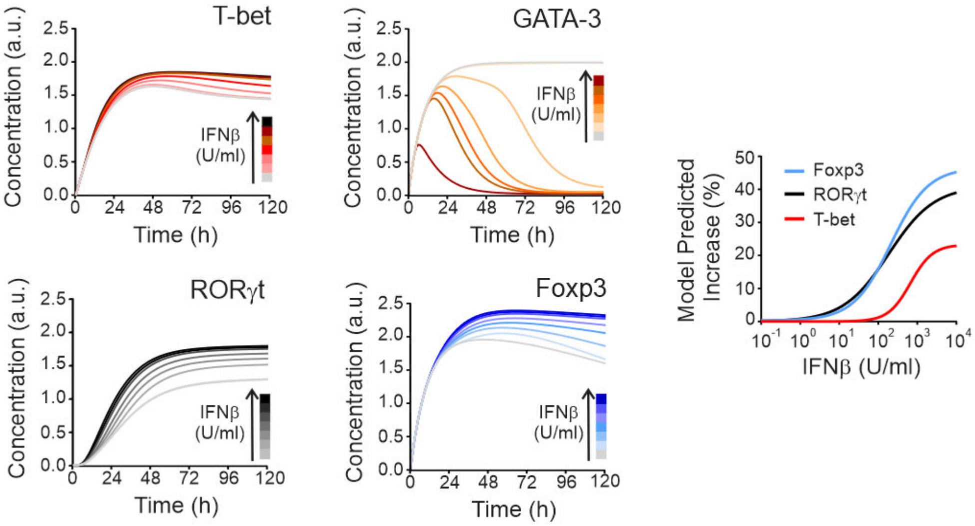

Figure 2. Maximal effects of IFNβ on T_H1, T_H2, T_H17 and T_{REG} polarizations utilizing an optimized coupling of IFNAR signaling to the STATs for each polarization. IFNβ concentrations varied from 0 to 10,000 U/ml for all simulations.

3. Calibration of the T_{REG}-optimized computational model to T_{REG} induction experimental data in the presence of IFNβ and IL-6

The calibration of the model was done in 3 steps.

1. We obtained the conversion of IFNβ concentration in U/ml to model units (mol/l). We performed proliferation experiments in $CD4^+$ Naïve T cells stimulated with αCD3/αCD28 in the presence of increasing concentrations of IFNβ. We simulated the model in the same conditions and obtained a dose-response-curve of the percentage of $IFN\gamma^+$ cells, assumed to be linearly related to the concentration of IFNγ in the model. We fit the simulated and the experimental dose-response and derived the mapping of IFNβ in experimental U/ml to mol/l. (**Fig.3 left**).
2. We simulated the T_{REG}-optimized model (see section 2) at 0, 10, 100, 1000 U/ml and compared the Foxp3 concentration at day 5 in the model with the experimental results expressed as the percentage of $Foxp3^+$ cells from **Figure 2C** in the main manuscript. We performed a linear regression to calibrate the T_{REG}-opt and extract the relationship between the concentration of Foxp3 in the model and the percentage of $Foxp3^+$ cells in a murine T_{REG} induction (**Fig.3 center**).
3. We proceeded to obtain the relationship between IL-6 concentration in the model and IL-6 concentration in the culture by fitting the percentage of $Foxp3^+$ cells in the model using the curve obtained in 2, to the T_{REG} induction experimental results obtained in the presence of IL-6 at 0.01 ng/ml, 0.1 ng/ml and 1 ng/ml (**Fig.3 right**).

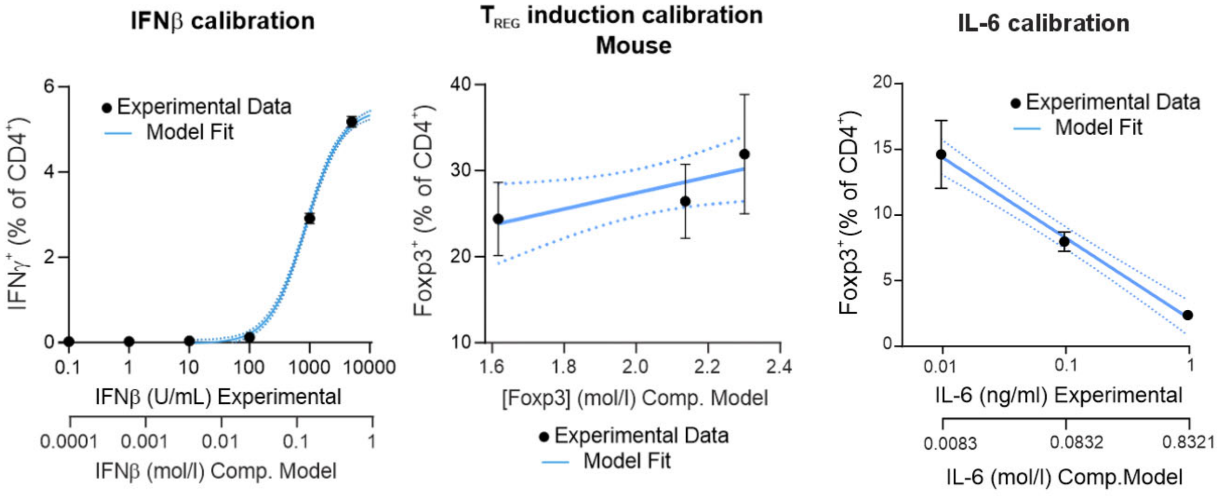

Figure 3. Calibration results of the computational model to experimental data.

References

1. Carbo A, Hontecillas R, Kronsteiner B, Viladomiu M, Pedragosa M, Lu P et al. Systems modeling of molecular mechanisms controlling cytokine-driven CD4+ T cell differentiation and phenotype plasticity. PLoS Comput Biol 2013;9(4):e1003027.

2. Fueyo-González F, McGinty M, Ningoo M, Anderson L, Cantarelli C, Andrea Angeletti et al. Interferon-β acts directly on T cells to prolong allograft survival by enhancing regulatory T cell induction through Foxp3 acetylation. Immunity 2022;55(3):459-474.e457.
3. Hoops S, Sahle S, Gauges R, Lee C, Pahle J, Simus N et al. COPASI--a COmplex PAthway SImulator. Bioinformatics 2006;22(24):3067-3074.
4. Platanias LC. Mechanisms of type-I- and type-II-interferon-mediated signalling. Nat Rev Immunol 2005;5(5):375-386.
5. van Boxel-Dezaire AH, Rani MR, Stark GR. Complex modulation of cell type-specific signaling in response to type I interferons. Immunity 2006;25(3):361-372.

Supplementary Table S1. Reagents

REAGENT or RESOURCE	SOURCE	IDENTIFIER
Antibodies		
PE-Cy7 anti-mouse CD4 antibody antibody	eBiosciences	Cat# 25-0041-82 RRID:AB_469576
PerCP Cy5.5 anti-mouse CD45 antibody	Invitrogen	Cat# 45-0451-82 RRID:AB_1107002
FITC anti-mouse CD3 antibody	Invitrogen	Cat# 11-0031-85 RRID:AB_464883
TIP60 (C-7) AF488	SantaCruz	Cat# Sc-166323
P300 (F-4) AF488	SantaCruz	Cat# Sc-48343
TIP60 (C-7) AF546	SantaCruz	Cat# Sc-166323
BV450 anti-human Foxp3	eBiosciences	Cat# 48-4776-42 RRID:AB_1834364
eFluor450 anti-mouse Foxp3	eBiosciences	Cat# 48-5773-82 RRID:AB_1518812
A488 anti-mouse Foxp3	eBiosciences	Cat# 53-5773-82 RRID:AB_1518812
FITC anti-mouse CD25 antibody	BD Biosciences	Cat# 553072 RRID:AB_394604
APC anti-IL17A antibody	eBiosciences	Cat# 17-7177-81 RRID:AB_763580
APC anti-mouse T-bet	eBiosciences	Cat#17-5825-82 RRID:AB_2744712
PE anti-human anti-mouse pSTAT1 (pY701) antibody	BD Biosciences	Cat# 612564; RRID:AB_399855
PacBlue anti-human anti-mouse pSTAT3 (pY705)	BD Biosciences	Cat# 560312; RRID:AB_1645327
αCD3 mouse	BioLegend	Cat# 100201 RRID:AB_312658
αCD3 human	BD Biosciences	Cat# 566685
αCD28 human	BD Biosciences	Cat# 555725
FITC anti-human Foxp3	eBiosciences	Cat# 11-4777-42 RRID:AB_1518812
PerCP Cy5.5 anti-human CD4 antibody	BD Biosciences	Cat# 552838 RRID:AB_394488
APC anti-human CD25 antibody	eBiosciences	Cat# 17-0259-42 RRID:AB_1582219
PE anti-human CD127 antibody	BD Biosciences	Cat# 557938 RRID:AB_2296056
FITC anti-human CD4 antibody	eBiosciences	Cat# 11-0042-86 RRID:AB_464898
Anti-Acetyl Lysine-HRP	Immunechem	Cat# ICP0381
Chemical, Peptides and Recombinant Proteins		
Recombinant murine IL-2	Peprotech	Cat# 212-12
Recombinant TGFβ1	Peprotech	Cat# 100-21C
Recombinant human IL-2	BD Pharmingen	Cat# 554603
CellTrace violet Cell Proliferation	Thermofisher	Cat# C34557
eFluor 780 Fixable viability dye	eBioscience	Cat# 65-0865-14
Recombinant murine IL-6	Peprotech	Cat# 216-16

Recombinant murine IFNβ	PBL Assay Science	Cat# 12410-1
Recombinant human IL-6	Peprotech	Cat# 216-06
Recombinant human IFNβ	PBL Assay Science	Cat# 11410-2
AffinityScript MultiTemp RT	Agilent	Cat# 600105
PlatinumTaq DNA polymerase	Invitrogen	Cat# 11804011
SYBR Green	Invitrogen	Cat# 4402959
TMP195	SelleckChem	Cat# S8502
Critical Commercial Assays		
EasySep™ Mouse Naïve CD4+ T Cell Isolation Kit	STEMCELL Technologies	Cat# 19765
EasySep™ Human Naïve CD4+ T Cell Isolation Kit	STEMCELL Technologies	Cat# 19555
EasySep™ Mouse T Cell Isolation Kit	STEMCELL Technologies	Cat# 19851
EasySep™ Mouse CD90.2 Positive Selection Kit II	STEMCELL Technologies	Cat# 18951
aCD3/aCD28 stimulating beads murine	Gibco	Cat# 11-161D
aCD3/aCD28 stimulating beads human	Gibco	Cat# 11-456D
Intracellular/transcription factor staining buffer kit	eBiosciences	Cat# 00-5523-00
